# Subsynaptic Domains in Super-Resolution Microscopy: The Treachery of Images

**DOI:** 10.3389/fnmol.2019.00161

**Published:** 2019-07-02

**Authors:** Xiaojuan Yang, Christian G. Specht

**Affiliations:** École Normale Supérieure, PSL Research University, CNRS, Inserm, Institute of Biology (IBENS), Paris, France

**Keywords:** subsynaptic domain (SSD), super-resolution microscopy, single molecule localization microscopy (SMLM), inhibitory receptors, gephyrin

## Abstract

The application of super-resolution optical microscopy to investigating synaptic structures has revealed a highly heterogeneous and variable intra-synaptic organization. Dense subsynaptic protein assemblies named subsynaptic domains or SSDs have been proposed as structural units that regulate the efficacy of neuronal transmission. However, an in-depth characterization of SSDs has been hampered by technical limitations of super-resolution microscopy of synapses, namely the stochasticity of the signals during the imaging procedures and the variability of the synaptic structures. Here, we synthetize the available evidence for the existence of SSDs at central synapses, as well as the possible functional relevance of SSDs. In particular, we discuss the possible regulation of co-transmission at mixed inhibitory synapses as a consequence of the subsynaptic distribution of glycine receptors (GlyRs) and GABA_A_ receptors (GABA_A_Rs).

**LAY ABSTRACT**

Super-resolution imaging strategies bypass the resolution limit of conventional optical microscopy and have given new insights into the distribution of proteins at synapses in the central nervous system. Neurotransmitter receptors and scaffold proteins appear to occupy specialized locations within synapses that we refer to as subsynaptic domains or SSDs. Interestingly, these SSDs are highly dynamic and their formation seems to be related to the remodeling of synapses during synaptic plasticity. It was also shown that SSDs of pre-and post-synaptic proteins are aligned in so-called nanocolumns, highlighting the role of SSDs in the regulation of synaptic transmission. Despite recent advances, however, the detection of SSDs with super-resolution microscopy remains difficult due to the inherent technical limitations of these approaches that are discussed in this review article.

## Introduction

Single molecule localization microscopy (SMLM) bypasses the diffraction limit by detecting signals from a sparse subset of molecules that are temporally separated, thus achieving a spatial resolution of single molecules of 10–40 nm (Schermelleh et al., 2010; Turkowyd et al., 2016; Sieben et al., 2018). SMLM includes several related techniques, namely STORM, PALM and uPAINT (Betzig et al., 2006; Hess et al., 2006; Rust et al., 2006; Giannone et al., 2010). In 2010, Dani et al. (2010) measured the laminar distribution of synaptic proteins using multicolor three-dimensional (3D) STORM, demonstrating the capability of SMLM to visualize the ultra-structure of synapses (Specht et al., 2014). This marks the beginning of super-resolution optical imaging of synaptic structures. Numerous studies have since applied SMLM to explore the heterogeneity and complexity of protein assemblies at synapses. Another type of super-resolution optical microscopy achieves sub-diffraction resolution by means of structured excitation, such as stimulated emission depletion (STED; Klar et al., 2000) and structured illumination microscopy (SIM; Gustafsson, 2000). Regardless of the different working principles, super-resolution microscopy techniques have yielded significant insights into the distribution of synaptic proteins on the nanometer scale. Given their wide-field, volumetric imaging strategies, three-dimensional and quantitative information can be gained from a large sample size.

In 2013, several groups reported independently that different synaptic proteins are distributed heterogeneously at synapses (MacGillavry et al., 2013; Nair et al., 2013; Specht et al., 2013). SMLM images showed that the excitatory scaffold protein PSD-95 occupies subdomains within the post-synaptic density (PSD) that regulate AMPAR clustering (MacGillavry et al., 2013; Nair et al., 2013). The existence of PSD-95 subdomains was confirmed with STED microscopy both *in vitro* and *in vivo* (Broadhead et al., 2016; Dzyubenko et al., 2016; Hruska et al., 2018; Masch et al., 2018; Wegner et al., 2018). Likewise, subsynaptic domains of gephyrin were shown to play a role in inhibitory plasticity at GABAergic synapses (Pennacchietti et al., 2017; Crosby et al., 2019). These findings point towards a mechanism whereby subsynaptic domains drive the recruitment of neurotransmitter receptors to specific locations within the PSD, thus regulating synaptic transmission.

SMLM and STED microscopy have also shown that pre-synaptic proteins of the active zone (AZ) and synaptic adhesion proteins display subsynaptic distributions (Perez de Arce et al., 2015; Chamma et al., 2016a,b; Tang et al., 2016; Glebov et al., 2017; Haas et al., 2018). Using multicolor 3D-STORM, Tang et al. (2016) demonstrated that subsynaptic domains of RIM1/2 are aligned with those of PSD-95, an arrangement that is referred to as trans-synaptic nanocolumn. The alignment of pre- and post-synaptic elements appears to be due to neuroligin/neurexin adhesion complexes (Perez de Arce et al., 2015; Haas et al., 2018). These exciting observations not only demonstrate the power of SMLM to visualize the ultra-structures of synapses but also point towards possible roles of subsynaptic domains in synaptic function (reviewed in Biederer et al., 2017; Liu et al., 2017; Chen et al., 2018; Scheefhals and MacGillavry, 2018).

Despite these advances, the concept of subsynaptic domains remains ambiguous, not least because the technical and biological limitations in identifying subsynaptic domains have not been sufficiently scrutinized. Here, we review the available evidence for the existence of subsynaptic domains, highlighting the factors that need to be taken into account in detecting small protein assemblies using SMLM. We then discuss the possible role of subsynaptic domains in the regulation of glycinergic and GABAergic co-transmission based on recent data from inhibitory synapses.

## What Is a Subsynaptic Domain?

### Terminology and Definition

A major source of confusion is that different names have been used in the literature to describe subsynaptic domains. Among these, the terms nanodomain, nanocluster, subcluster, subdomain and nanomodule have been used in an interchangeable manner (e.g., MacGillavry et al., 2013; Nair et al., 2013; Broadhead et al., 2016; Haas et al., 2018; Hruska et al., 2018). The lack of a clear and unified terminology has made it difficult to refer to specific molecular structures and to be aware of the differences and similarities between studies. Regarding the choice of words, the term *cluster* should best be avoided, because it can also refer to the clustering algorithms that are widely used for image analysis of SMLM data (Nicovich et al., 2017). The prefix *nano* is redundant because synapses themselves have diameters of only a few hundred nanometers. Furthermore, *nanodomain* has been widely used to describe the high Ca^2+^ ion concentrations in the proximity of an open calcium channel (Augustine et al., 2003; Eggermann et al., 2013; Ghelani and Sigrist, 2018).

We, therefore, refer to these structures as *subsynaptic domain* or *SSD* (Crosby et al., 2019) for the following reasons: (1) the term is self-explanatory, referring to a space that is smaller than the whole synaptic compartment and that is occupied by a given type of molecules; and (2) it is flexible in that it can be equally applied to membrane receptors, scaffold and signaling proteins, whether they are pre-synaptic or post-synaptic. We define SSD as a sub-compartment of the synapse in which the density of a specific synaptic protein is higher than in the surrounding area, and that is typically observed with super-resolution microscopy. We believe that the term SSD could thus provide some clarity in defining specific molecular entities at synapses.

### SSD Size and Protein Copy Numbers

The most basic feature of SSDs that holds biologically relevant information is their size and the copy number of proteins that they contain. A wide range of sizes was detected by SMLM and STED microscopy (Table 1). For instance, SSDs of excitatory scaffold proteins in cultured hippocampal neurons have a diameter of ~80 nm as judged by coordinate-based SMLM analysis (MacGillavry et al., 2013), whereas an average diameter of 120 nm was measured in reconstructed super-resolution images (Nair et al., 2013). STED microscopy detected SSDs of PSD-95 with a diameter of 200 nm (Fukata et al., 2013). These differences in SSD size are likely due to the different resolution of the imaging systems and the application of a threshold during image processing. A comparative study of PSD-95 in hippocampal tissue using PALM and STED determined median SSD diameters of 126 nm and 158 nm, respectively, exemplifying the impact of the imaging approach (Broadhead et al., 2016). The typical diameter of the whole PSD in hippocampal neurons ranges from 100 nm to 800 nm, with a mean of about 300 nm (Harris and Stevens, 1989; Arellano et al., 2007). Therefore, the lower limit of SSD sizes of ~50 nm reflects the image resolution of the super-resolution imaging techniques, while the upper limit corresponds to the size of the entire synapse. Given that synapse sizes vary substantially across the central nervous system, an interesting question is whether SSDs of different synaptic proteins have stereotypical sizes that are the same at different types of synapses (see Crosby et al., 2019).

**Table 1:** Size and protein copy numbers of SSDs and PSDs obtained with different experimental techniques.

Structure	Diameter (nm)	Molecule numbers	Technique	Synapse type	References
SSD	50–130*		SMLM	Excitatory, hippocampal	MacGillavry et al. (2013), Nair et al. (2013), Broadhead et al. (2016), Chamma et al. (2016a,b) and Haas et al. (2018)
	130–760*		STED	Excitatory, hippocampal and cortical	Nair et al. (2013), Broadhead et al. (2016) and Hruska et al. (2018)
	~300*		SIM	Inhibitory, hippocampal	Crosby et al. (2019)
	70*	~20 AMPARs/SSD*	STORM	Excitatory, hippocampal	Nair et al. (2013)
PSD	300 (100–800)^#^		EM	Excitatory, hippocampal	Harris and Stevens (1989) and Bourne and Harris (2011)
	290 (110–650)^#^		EM	Excitatory, cortical	Arellano et al. (2007) and Santuy et al. (2018)
	350 (110–700)^#^		EM	Inhibitory, hippocampal and cortical	Bourne and Harris (2011) and Santuy et al. (2018)
		50 (0–200) AMPARs^#^	EM	Excitatory, various CNS regions	Masugi-Tokita et al. (2007), Tarusawa et al. (2009) and Fukazawa and Shigemoto (2012)
		30 (0–200) GABA_A_Rs^#^	Electrophysiology, EM	Inhibitory, cerebellar and hippocampal	Nusser et al. (1997, 1998)
		30 (40–500) PSD-95^#^	Biochemistry, TIRF microscopy	Excitatory, various brain regions	Sugiyama et al. (2005) and Sheng and Kim (2011)
		30 (40–500) Gephrin^#^	SMLM (decay recordings)	Inhibitory, spinal cord	Specht et al. (2013) and Patrizio et al. (2017)

*Mean values obtained in the cited studies; ^#^mean (range in brackets). Values were taken directly or calculated from those reported in the cited studies.

Information about protein copy numbers is essential to establish the structural basis of SSD formation. To date, there are hardly any quantitative data about SSD molecule numbers. SSDs of AMPARs have been estimated to contain an average of ~20 receptor complexes (Nair et al., 2013). Due to the limited accessibility of the epitopes for immunolabeling, however, the actual number of receptors per SSD could be higher. This could have an effect on the role of SSDs in synaptic function since the number of active receptors is directly related to the strength of synaptic transmission (Masugi-Tokita et al., 2007; Tarusawa et al., 2009; Fukazawa and Shigemoto, 2012).

### Number of SSDs Per Synapse

Most synapses contain only one SSD or no SSD at all. More specifically, a single SSD was detected in 50% to 80% of synapses imaged with SMLM, SIM or STED microscopy, less than 20% had more than three SSDs, and six SSDs was the upper limit (MacGillavry et al., 2013; Nair et al., 2013; Broadhead et al., 2016; Chamma et al., 2016a,b; Pennacchietti et al., 2017; Hruska et al., 2018; Crosby et al., 2019). It is likely that the different imaging techniques and analyses again have an effect on the detection of multiple SSDs. This raises the question whether the SSD simply reflects the center of mass of the protein assembly, and if so, whether the presence of single or multiple SSDs actually matter for the regulation of synaptic function.

There exists a positive correlation between the number of SSDs and the size of the PSD or the dendritic spine (Fukata et al., 2013; Nair et al., 2013; Hruska et al., 2018; Crosby et al., 2019). EM studies have revealed a large variability in PSD area, ranging from 100 nm to 800 nm in diameter (Table 1). More than half of the PSDs are small (<0.05 μm^2^), which is similar to the fraction of synapses with only one SSD (Arellano et al., 2007). Moreover, the number of AMPAR molecules is positively correlated with the PSD size, and large complex PSDs have a higher density of AMPARs than small, non-perforated PSDs (Ganeshina et al., 2004; Shinohara et al., 2008; Fukazawa and Shigemoto, 2012). Together, these data indicate that SSDs may only play a role at large PSDs, reflecting the superior strength of these synapses.

### Trans-synaptic Nanocolumns

From the viewpoint of neuron connectivity, pre-synaptic and post-synaptic SSDs can be aligned to form trans-synaptic structural units that regulate synaptic function (Biederer et al., 2017; Chen et al., 2018). Such an organization has been observed at excitatory synapses using 3D-SMLM, and was suitably named *trans-synaptic nanocolumn* (Tang et al., 2016). SMLM studies have further shown that synaptic adhesion complexes such as neuroligin and neurexin are also organized in SSDs, suggesting that they contribute to the formation of trans-synaptic nanocolumns (Perez de Arce et al., 2015; Haas et al., 2018). The term nanocolumn, therefore, refers to a specific concept, namely the alignment of pre- and post-synaptic SSDs that brings together different functional elements. Future studies are expected to explore the possible role of nanocolumns in synaptic plasticity.

### The Dynamics of SSDs

The hypothesis that SSDs regulate synaptic transmission implies that SSDs adapt dynamically to changes in synaptic strength. Indeed, live SMLM in cultured neurons has revealed the mobility and morphological changes of SSDs. Synaptic scaffolds undergo dynamic changes on a timescale of 5–10 min, displaying marked differences in the number, position and shape of SSDs at different time points (Nair et al., 2013; Specht et al., 2013; Rodriguez et al., 2017). STED microscopy further showed that these morphological changes occurred both *in vitro* and *in vivo* (Hruska et al., 2018; Wegner et al., 2018). The dynamics of SSDs are in agreement with the exchange of individual proteins at synaptic and extra-synaptic sites, which is a hallmark of the dynamic synapse (Choquet and Triller, 2013; Delgado and Selvin, 2018). Therefore, SSDs are momentary representations of the protein distribution and need to be viewed as dynamic snapshots rather than rigid structural units.

## How to Detect Subsynaptic Domains With Smlm

The identification of SSDs consists in detecting small numbers of densely packed molecules in a confined space with a high local background from neighboring molecules with lower density. Despite these challenges, SMLM is well suited to resolve the internal organization of small structures such as synapses at single molecule level. In the following, we discuss the relevant factors of the image acquisition and data analysis that have an impact on the identification of SSDs.

### Image Acquisition

SMLM techniques aim to record large numbers of single fluorophore detections from densely labeled structures, while ensuring that the signals are sufficiently sparse to be well separated. STORM, PALM and uPAINT have all been employed for detecting SSDs. The three techniques have the same intrinsic challenges when it comes to the ultrastructure of synapses, chief among them being the fluorophore. Most fluorophores are detected repeatedly due to their fluorescence lifetime, photo-switching and blinking. This can create dense clusters of redundant detections that are easily mistaken for SSDs. The blinking behavior of the fluorophores (organic dyes or fluorescent proteins) is dependent on their photo-physical and photo-chemical properties, and it can be modulated by the laser power and the composition of the imaging buffer (Dempsey et al., 2011; Endesfelder et al., 2011; van de Linde et al., 2011; Nahidiazar et al., 2016). Sub-optimal imaging conditions such as inefficient laser illumination or an incompatible buffer system can result in artificial clustering (Annibale et al., 2011; Burgert et al., 2015; Nahidiazar et al., 2016). Even with an optimized imaging protocol, different fluorophores will produce different representations of the analyzed structure (Dempsey et al., 2011; Baddeley and Bewersdorf, 2018). The evaluation of the number and the size of SSDs is therefore strongly dependent on the fluorophores, and control experiments with different fluorophores are crucial to validate the experimental findings (Yang and Specht, in press). In addition to the fluorophores, attention should also be drawn to the labeling strategies used for sample preparation. The distance between the fluorophores and the actual positions of the target molecules (e.g., due to the size of antibodies used for labeling), and under-sampling due to a limited labeling efficiency can add to the uncertainties in the identification of SSDs (Deschout et al., 2014; Maidorn et al., 2016).

### Image Segmentation

Depending on the type of SMLM data (pointillist or reconstructed super-resolution images), different algorithms have been adopted for segmenting SSDs. For coordinates-based data, a local density threshold is generally applied. The local density can for instance be defined as the number of detections within a radius of five times the mean nearest neighbor distance of all the detections within each synapse, and SSDs are identified as regions above a certain threshold (MacGillavry et al., 2013; Tang et al., 2016; Pennacchietti et al., 2017). As regards the reconstructed images, an intensity threshold may be adopted instead. For example, wavelet segmentation has been used to identify SSDs at synapses in the whole field of view (Nair et al., 2013; Chamma et al., 2016a,b). Similarly, watershed segmentation can be employed to segment SSDs of individual synapses in reconstructed SMLM images or deconvoluted STED images (Broadhead et al., 2016; Dzyubenko et al., 2016). The difficulty of all these approaches is that the detected size and the number of SSDs are directly dependent on the algorithms and the chosen parameters, which makes an accurate identification of SSDs challenging.

### Dealing With Small Molecule Numbers and the Variability of Synapses

Synapses exhibit a large variability not only in size but also in terms of molecule numbers. Neurotransmitter receptors such as AMPARs or GABA_A_Rs have relatively low copy numbers, with an average of ~50 receptor complexes per synapse (ranging up to 200 copies; Table 1). The main scaffold proteins at excitatory and inhibitory synapses outnumber the receptors by a factor of four to five. PSD-95 and gephyrin molecules amount to 40–500 per synapse, with an average of ~300 copies (Sugiyama et al., 2005; Sheng and Kim, 2011; Specht et al., 2013; Patrizio et al., 2017). The low copy numbers of synaptic proteins, especially receptors, makes the identification of SSDs with SMLM challenging since the labeling of the structures is often rather faint. At the same time, the high local density of synaptic proteins can further reduce the efficiency of immunolabeling due to epitope masking. The overall receptor density at synapses is in the order of 700 AMPARs/μm^2^ for the whole PSD (50 AMPARs/0.07 μm^2^). An average SSD with a diameter of 70 nm (area of 0.0038 μm^2^) contains about 20 AMPARs, resulting in an estimated density of ~5,000 AMPAR complexes/μm^2^ (Nair et al., 2013). Considering the molecular size of the receptor complexes (10 nm × 20 nm; Patriarchi et al., 2018), 20 AMPARs would occupy a membrane area of at least 0.004 μm^2^. This means that the receptors are very densely packed inside the SSD, adding to the uncertainties that result from the stochasticity of the immunolabeling and fluorophore detection.

### Alternative Approaches

Given the rapid advances in super-resolution imaging technologies, promising alternatives for the investigation of complex structures such as synapses are quickly emerging. Among these, smaller probes such as nanobodies have been produced to bypass the limitations of labeling density and to minimize the distance between the fluorophores and the target proteins (Chamma et al., 2016a; Maidorn et al., 2016). DNA-PAINT allows multi-color SMLM imaging (Nieves et al., 2018). DNA origami standards provide a more precise way for calibrating protein copy numbers given that the absolute quantification of molecules at SSDs is faced with large stochasticity of the imaging technique (Zanacchi et al., 2017). Furthermore, new algorithms are being developed to segment synaptic clusters in coordinates-based datasets more efficiently (Nicovich et al., 2017; Baddeley and Bewersdorf, 2018).

## The Emerging Role of Ssds in Inhibitory Synaptic Transmission

**Figure 1:**
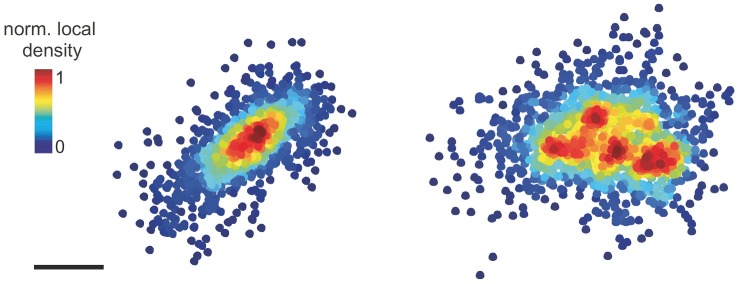
Pointillist images showing synaptic gephyrin clusters with one SSD (left) or four SSDs (right). The points represent the detections of single fluorophores from PALM imaging. Scale bar: 100 nm (adapted with permission from Pennacchietti et al., 2017).

Electron microscopy of symmetric synapses has revealed a discontinuous network of filaments at the inhibitory PSD and in the synaptic cleft (Linsalata et al., 2014; High et al., 2015). Super-resolution optical microscopy confirmed that the inhibitory scaffold protein gephyrin forms synaptic clusters of variable morphology that can undergo dynamic changes and may contain SSDs (Specht et al., 2013; Dzyubenko et al., 2016; Pennacchietti et al., 2017; Crosby et al., 2019). SMLM imaging in cultured hippocampal neurons further revealed that extra-synaptic gephyrin molecules are recruited to synaptic sites during NMDA-induced inhibitory long-term potentiation (Pennacchietti et al., 2017). The increase in molecule density was accompanied by an increased fraction of gephyrin clusters with multiple SSDs (Figure 1). More recently, Crosby et al. (2019) conducted a comprehensive analysis of pre- and postsynaptic components using 3D-SIM, reaching a resolution of ~120 nm laterally and ~300 nm axially. It was shown that GABA_A_Rs form SSDs with an average diameter of ~300 nm that are closely associated with SSDs of gephyrin and pre-synaptic RIM (Crosby et al., 2019). This implies the existence of trans-synaptic nanocolumns as an organizing principle of inhibitory synapses. Given that the measured size of the SSDs was close to the resolution limit, the concept of nanocolumns at inhibitory synapses will require further validation. Nonetheless, these studies strongly suggest that the internal organization of inhibitory synapses plays an important role in regulating synaptic transmission.

Unlike the cortex and hippocampus where fast neuronal inhibition is mainly mediated by GABA_A_Rs, both glycine and GABA receptors coexist at synapses in the brainstem and the spinal cord. Gephyrin provides binding sites for the immobilization of both types of receptor (reviewed in Choii and Ko, 2015; Alvarez, 2017; Groeneweg et al., 2018; Specht, 2019). Several GABA_A_R subunits bind to gephyrin, albeit with a lower affinity than the GlyRβ subunit (e.g., Maric et al., 2011; Kowalczyk et al., 2013). We do not yet know whether GlyRs and GABA_A_Rs form SSDs at mixed synapses, and if so, how they are related to the SSDs of gephyrin. Mixed inhibitory synapses are activated by the co-release of glycine and GABA from presynaptic vesicles (Jonas et al., 1998; Aubrey and Supplisson, 2018). This creates a situation, where the exact position of GlyRs and GABA_A_Rs relative to the pre-synaptic release site can have a strong impact on the efficacy of the agonists and thus the activity of the receptors. Through its capacity to resolve the spatial organization of mixed inhibitory synapses, SMLM may provide answers to these open questions.

## Outlook

The concept of SSDs as dynamic units underlying synaptic strength provides a new angle to interpret the function of synapses. SMLM and other super-resolution imaging techniques are powerful tools to investigate the internal organization of synapses. Given the intrinsic stochasticity of SMLM and the inherent variability of synaptic protein assemblies, however, the identification and characterization of SSDs demand great scrutiny in the experimental and analytical procedures. Super-resolution techniques may still have some way to go before we can truly resolve the fast molecular processes at synapses.

## Author Contributions

All authors listed have made a substantial, direct and intellectual contribution to the work, and approved it for publication.

## Conflict of Interest Statement

The authors declare that the research was conducted in the absence of any commercial or financial relationships that could be construed as a potential conflict of interest.
